# Light-weight neural network for intra-voxel structure analysis

**DOI:** 10.3389/fninf.2024.1277050

**Published:** 2024-09-09

**Authors:** Jaime F. Aguayo-González, Hanna Ehrlich-Lopez, Luis Concha, Mariano Rivera

**Affiliations:** ^1^Centro de Investigacion en Matematicas, Guanajuato, Mexico; ^2^Department of Behavioral and Cognitive Neurobiology, Institute of Neurobiology, National Autonomous University of Mexico, Queretaro, Mexico

**Keywords:** intra-voxel structure, DW-MRI, neural network, deep learning, self-supervised learning, fixels

## Abstract

We present a novel neural network-based method for analyzing intra-voxel structures, addressing critical challenges in diffusion-weighted MRI analysis for brain connectivity and development studies. The network architecture, called the Local Neighborhood Neural Network, is designed to use the spatial correlations of neighboring voxels for an enhanced inference while reducing parameter overhead. Our model exploits these relationships to improve the analysis of complex structures and noisy data environments. We adopt a self-supervised approach to address the lack of ground truth data, generating signals of voxel neighborhoods to integrate the training set. This eliminates the need for manual annotations and facilitates training under realistic conditions. Comparative analyses show that our method outperforms the constrained spherical deconvolution (CSD) method in quantitative and qualitative validations. Using phantom images that mimic *in vivo* data, our approach improves angular error, volume fraction estimation accuracy, and success rate. Furthermore, a qualitative comparison of the results in actual data shows a better spatial consistency of the proposed method in areas of real brain images. This approach demonstrates enhanced intra-voxel structure analysis capabilities and holds promise for broader application in various imaging scenarios.

## 1 Introduction

The study of neural structure using diffusion-weighted (DW) magnetic resonance imaging (MRI) is relevant for connectivity research and clinical applications. One can infer the local white matter structure by measuring the DW signals along multiple directions. These measurements contribute to the study of brain connectivity patterns and the detection of some brain diseases. For example, the information on the local diffusion directions describing the tissue structure is essential for constructing a diffusion tractography brain model (Nucifora et al., 2007). In diffusion tractography, the method used to infer the intra-voxel structure plays an important role in the quality of the estimation of the anatomy of the human brain (Schilling et al., 2021). In addition, DW imaging is useful for the detection of ischemic stroke, trauma, and brain tumors (Gaddamanugu et al., 2022).

Many models with different characteristics have been developed to recover orientation information from the microstructure of brain tissue. Among them, diffusion tensor imaging (DTI) is one of the most straightforward approaches, based on the Gaussian diffusion model for water movement in biological tissues (Basser, 1995; Soares et al., 2013). DTI approximates the diffusion propagator by a 3–variate normal distribution with a mean of zero, modeled by the diffusion tensor (DT) (Basser, 1995). This model is sound for signals acquired from a single coherently oriented fiber; however, the model is too simplistic for modeling more complex fiber configurations. This is important as ~60 to 90% of diffusion data voxels have fibers that cross, kiss, fan, or bend (Jeurissen et al., 2013), limiting the capabilities of DTI in accurately estimating the microstructure in realistic scenarios. Because of this limitation, several methods have been developed to model the complex fiber configurations of more than one axonal bundle.

Some notable examples of multi-tensor (MT) modeling include diffusion multi-tensor (DMT) modeling for a finite number of orientations (Tuch et al., 2002), Q-ball modeling to reconstruct the diffusion orientation distribution function (Tuch, 2004), constrained spherical deconvolution (CSD) to reconstruct the fiber orientation distribution function (fODF) (Tournier et al., 2007), and non-negative least squares (Ramirez-Manzanares et al., 2007). While DMT generalizes DTI for more than one fiber, the estimation of the fODF via CSD adheres to a non-parametric method. These methods rely on an optimization problem to determine the combination of signals from a dictionary that better reconstructs the measured signal (Tournier et al., 2007). Non-parametric models exhibit more reliability in voxels with crossing fibers (Jeurissen et al., 2011) and depend on fewer parameters than DMT. For these reasons, CSD has been established as one of the most used methods for intra-voxel structure analysis.

However, there are some disadvantages in using traditional methods for intra-voxel structure analysis. For example, CSD is known to provide an overestimation of the number of fibers and it tends to be inaccurate in data with high levels of noise (Jeurissen et al., 2014). To overcome some issues, some improvements to CSD have been proposed. Deep neural networks (DNNs) have recently become a rapidly growing subset of machine learning algorithms that automatically learn the results of interest from data rather than hand-crafted features (Latha et al., 2021). These methods are used to learn models that map the diffusion signal to specific diffusion parameters. Recent studies have shown that DNNs can be competitive with state-of-the-art techniques, improving in areas such as the number of signal acquisitions required for a good estimation, computational complexity, and precision of estimates. Some examples of these methods are LSTM units to extract features as the volume fractions of different compartments (Ye et al., 2019), a U-Net to generate the fractional anisotropy, the mean diffusivity and the fiber tractography (Li et al., 2021), and a multi-layer perceptron (MLP) to address the intricated task of mapping diffusion-weighted signals onto the target fODF (Karimi et al., 2021). In addition, a previous study (Ehrlich and Rivera, 2021) explores the multi-layer perceptron, AxonNet, to estimate the brain nerve bundle orientations and volume fractions for a voxel using data from a small neighborhood around that voxel.

Motivated by the success of DNNs in DW analysis, we propose a novel deep neural network architecture for estimating the orientations and volume fractions of axonal bundles. Our model is based on a self-supervised learning approach: our non-parametric method is implemented by a deep neural network trained with noisy synthetic data. A key feature of our model is the exploitation of spatial correlation between neighboring voxels to improve inference and reduce the number of parameters required. Our model's estimation of orientations and volume fractions achieves competitive results compared to CSDs. It improves the estimation accuracy of images with a high noise level and the angle resolution of the estimated orientations in images with few signals. In addition, for evaluation purposes, we propose using a distance initially introduced in the context of Computational Optimal Transport: the Earth Mover's Distance (EMD) (Monge, 1781). We also discuss the convenience of using EMD over other metrics proposed in the literature. Through some experiments, we show the performance of our model and compare it to CSD using these metrics.

Table 1 lists the acronyms used in this study.

**Table 1:** Acronyms used in this article.

CSD	Constrained spherical deconvolution
DW	Diffusion-weighted
MRI	Magnetic resonance imaging
DTI	Diffusion tensor imaging
DT	Diffusion tensor
DMT	Diffusion multi-tensor
MT	Mutli-tensor
DNN	Deep neural network
MLP	Multi-layer perceptron
EMD	Earth mover's distance
NNLS	Non-negative least squares
CNN	Convolutional neural network
fODF	Fibers orientation distribution Function
SNR	Signal-to-noise ratio
MSE	Mean squared error
HARDI	High angular resolution diffusion imaging
SR	Success rate
GRP	Global relative performance
LNNN	Local neighborhood neural network

## 2 Notation and problem definition

A DW image is a three-dimensional (3D) array of spatially related signals that we denote by **S**. Each signal, *S* ∈ **S**, is a vector of size *n*, which is assumed to be a sample of a diffusion model. Each entry, *S*_*i*_, is associated with a gradient direction vector *g*_*i*_ and a scalar *b*-value *b*_*i*_. The gradient direction vectors are unitary; that is, |*g*_*i*_| = 1, *i* = 1, …, *n*, and the *b*-values are scalars that depend on the strength, duration, and spacing of the pulsed gradients in the DW acquisitions. The choice of the number of acquisitions, *n*, the gradient directions and their associated *b*-value, ${\left\{g_{i},b_{i}\right\}}_{i=1}^{n}$, is known as the *acquisition protocol* of the image. We will use the set notation $G={\left\{\left(g_{i},b_{i}\right)\right\}}_{i=1}^{n}$ for its representation.

For a given measure, *S* ∈ ℝ^*n*^ in **S**, our interest is to provide information about the tissue's microstructure corresponding to this signal. In particular, we characterize the structure by determining the number of axonal bundles, their orientations, and their contribution to generating the signal *S*. We denote these values as *k*, ${\left\{d_{i}\right\}}_{i=1}^{k}$, and ${\left\{\alpha_{i}\right\}}_{i=1}^{k}$, respectively. We usually use the term *fibers* to refer to the axon bundles and their contributions to the signal as *volume fractions*. For the estimation of these data, we assume that there exists a function, *F*, so that


$$\begin{array}{l} S=F\left(\left\{\alpha_{i}\right\},\left\{d_{i}\right\};G,\epsilon \right), \end{array}$$

for some random noise, ϵ, usually modeled through a Rician distribution (Gudbjartsson and Patz, 1995). Unfortunately, the transition from *S* to {α_*i*_}, {*d*_*i*_} is an ill-posed problem.

### 2.1 Brief review of theoretical models

For a voxel with a single fiber, the preeminent approach used to delineate *F* is the widely embraced diffusion tensor (DT) model, as documented in the study of Basser (1995). The DT is defined as


$$S(g_{i},b_{i})=S0e(-b_{i}g_{i}^{⊤}Dg_{i});$$

in which the unknown variable *D* ∈ ℝ^3 × 3^ represents the covariance matrix of diffusivity. At the same time, *S*0 denotes the measured signal obtained without diffusion weighting (i.e., when the *b*-value is zero). The fiber orientation is recovered from this model by computing the larger eigenvector of *D*; that is, the eigenvector associated with the larger eigenvalue. This vector signifies the orientation of higher diffusivity and is expected to be aligned with the fiber orientation.

As mentioned in the introduction, according to some authors (Ferizi, 2014), most of the diffusion data correspond to signals of more than one fiber crossing. To model these complex signals, numerous methods have been introduced; for example, DMT (Tuch et al., 2002), CSD (Tournier et al., 2007), and NNLS (Ramirez-Manzanares et al., 2007). They can be broadly categorized into two main types: parametric and non-parametric approaches.

A popular example of a parametric model is the **diffusion multi-tensor (DMT)** model (Tuch et al., 2002). DMT is a linear combination of *t* simple diffusion tensors in the same model, each with its corresponding parameters, that model a signal of crossing fibers. The DMT model is expressed as follows:


$$\begin{array}{l} S\left(gi,bi\right)=S_{0}\overset{t}{\underset{j=0}{\sum }}\left(-b_{i}g_{i}^{⊤}D_{j}g_{i}\right). \end{array}$$

In the case of non-parametric approaches, two of the most popular methods are **non-negative least square (NNLS)** (Ramirez-Manzanares et al., 2007) and **constrained spherical deconvolution (CSD)** (Tournier et al., 2007). Both are based on an optimization problem to determine the combination of signals from a dictionary that better reconstructs the original signal. This dictionary can approximate the orientation and volume fraction of the underlying fibers.

Formally, the NNLS problem can be defined as follows:


$$\begin{array}{l} x^{*}=\arg\underset{x}{\min}∥Ax-y{∥}^{2}\text{subject to }x\geq 0 \end{array}$$

where *A* ∈ ℝ^*m*×*n*^ is the fixed dictionary of diffusion signals, *y* is the data vector, and x^*^ ∈ ℝ^*m*^ is our vector solution.

The original CSD method (Tournier et al., 2007) assumes that the diffusion signal within a voxel can be modeled as a sum of spherical functions, each representing the contribution of a different fiber orientation. These spherical functions are convolved with a response function characterizing the point spread function of the image acquisition. The estimation process involves solving a sequential quadratic objective minimization problem:


$$\begin{array}{l} x^{t+1}=\arg\underset{x}{\min}∥Ax-y{∥}^{2}+\lambda ||L^{⊤}x|{|}^{2}. \end{array}$$

In this formulation, *x* represents the fODF coefficients, *y* is the observed diffusion signal, *A* represents a linear combination of spherical basis functions (often using spherical harmonics of order zero), and *L* is a penalization matrix that penalizes negative contributions of the estimated signal at each direction of the basis functions. The objective function minimizes the difference between the estimated signal (*Ax*) and the observed signal (*y*) while applying a regularization term to enforce constraints on the estimated fODF. The regularization parameter λ controls the strength of the regularization.

## 3 Materials and methods

In this study, we propose a non-parametric method for intra-voxel structure analysis. It consists of a model that infers the number of fibers, their orientations, and their volume fractions. For this purpose, we propose to employ a neural network whose expected output is interpreted as a discretized fODF over a dictionary D of *d* orientations. The network is trained using synthetic data generated by the DTM model, and the training is self-supervised. This section describes the model's architecture, the synthetic data generation, and the training procedure.

### 3.1 Model overview

Our model builds upon four fundamental principles, each contributing to its effectiveness. First, it exploits the additional information derived from neighboring signals to enhance the prediction accuracy of a voxel's microstructure. Second, it incorporates a specialized architecture designed to process neighboring signals while maintaining reasonable trainable parameters. Third, we introduce a procedure for generating a synthetic dataset that is simple but realistic enough to ensure the neural network's correct training. Finally, encoding the orientations and volume fractions to create the ground truth targets facilitates our network's seamless training.

As previously mentioned, our model exploits the spatial correlation of neighboring voxels. Two observations support the idea of using this additional information. First, the fiber orientations are expected to change moderately in neighboring voxels, as suggested in various tractography studies (Nucifora et al., 2007). Second, if the noise in each voxel is assumed to be uncorrelated (Salvador et al., 2005), different noise levels are present in each voxel, altering the inference. Therefore, a group of neighboring signals around a central voxel could provide more information about the orientation of the fibers and effectively average out the noise, improving the structure's inference.

It is important to note that ours is not the first method to consider using information from adjacent voxels for this task. For instance, Lin et al. (2019) proposed a model incorporating first-order neighboring voxels for inference. Furthermore, a previous study (Ehrlich and Rivera, 2021) showed that incorporating neighborhood information as input for multi-layer perceptrons improves prediction quality. In light of these findings, we have designed our neural network to accept a 3 × 3 × 3 voxel patch as input, enabling the inference of the structure of the central voxel within the patch. Concerning the neighborhood size, one could be tempted to define a larger neighborhood as this incorporates more information into the model; however, increasing the size compromises the network's efficiency, and this new information might not improve the result. Thus, we decided to use the smallest neighborhood that could contain all the possible orientations the fibers in the central voxel can take, covering a volume of 0.216*cm*^3^.

Despite the benefits of considering more voxels to improve the inference, this decision comes with the trade-off of processing a larger volume of signals. In our case, the input data expand from a single voxel to 27 voxels. This expansion also impacts the design of the neural network as a change in the input data size typically necessitates an increment in the number of neurons in subsequent layers (Xu and Chen, 2008). For example, Ehrlich's neighborhood configuration of the AxonNet (Ehrlich and Rivera, 2021) employs a multi-layer perceptron (MLP) with seven layers and nearly 20 million parameters to handle the increased input size. To tackle this challenge, we can use other types of architecture more appropriate for spatially correlated data.

One approach that tackles this drawback is to use a convolutional neural network (CNN). Some studies show experiments using this type of network (Lin et al., 2019; Aliotta et al., 2021). However, the convolutional layers impose a strong assumption about the relationship between neighboring signals and the structure we want to uncover. The convolutions are linear operators on the underlying data, designed to learn low levels of abstraction. As we know nothing about how the relationship of the signals can be uncovered, we believe that replacing the simple convolutions with a more expressive non-linear function can improve the local model's abstraction capability.

### 3.2 Neural network architecture

In this study, we introduce a novel architecture to solve the problem. Our proposal uses the same parameter-sharing scheme of CNNs but follows the idea proposed by Lin et al. (2013). This relies on the same assumption as CNNs: If one feature is useful for inferring the structure at some spatial position, it should also be useful at a different position. However, instead of convolutional layers, we use perceptrons: a well-studied function approximator (Mcculloch and Pitts, 1943). With this change, we assume nothing about the type of relationship between neighboring voxels that is relevant to infer the intra-voxel structure. Nevertheless, as with CNNs, the same dense network is shared between regions of voxels by dragging it over the input data.

**Figure 1:**
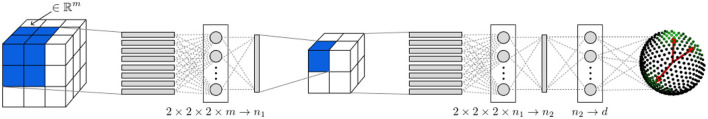
Diagram showing our NN's architecture proposal and how it evaluates a datum, represented as a cube of voxels.

The network consists of three layers as illustrated in Figure 1. We adopt the idea of Lin et al. (2013) in the first two layers of the network. The first layer is dense with ReLU activation functions, fed by a chunk of 2 × 2 × 2 voxels. The output of this layer is a vector of size *n*_1_ that we interpret as a feature vector, or descriptor, of the input neighborhood. As the full patch taken by the model is a cube of 3 × 3 × 3 voxels, each with *m* signals, it is possible to take eight blocks of size 2, each one taking a different corner, to be processed by the first layer, as it is portrayed in the first part of Figure 1. Following this procedure, after the first layer, we get eight vectors that we arrange graphically as a cube of size 2 × 2 × 2, with each voxel of size *n*_1_. Note that the eight descriptors obtained from the first layer were built by considering 2-voxel-sized neighborhoods containing the central voxel. Therefore, we expect the descriptors to include structural information of the central voxel based on the direction toward which the neighboring voxels are biased. We think this information should help the model to infer the struture of the central voxel.

For those familiar with convolutional neural networks, the evaluation mechanism of the first layer can be expressed in convolutional terms. This first part of the network can be seen as a 3D-convolutional layer that uses *n*_1_ filters of size (2, 2, 2) to process data of size (3, 3, 3), with *m* channels (the number of signals), at a stride of 1. The output is then evaluated in a ReLU activation function. The second layer of the architecture works as the first one with a few changes. This layer takes the descriptors generated in the first layer. Still, as there is only one chunk of size 2 × 2 × 2, the output is a vector of length *n*_2_ that encompasses the information needed for the inference (see the middle part of Figure 1). As in the previous layer, ReLU is used as an activation function. The resulting vector is then passed to a linear layer and evaluated in a Softmax activation function. The inference consists of a vector of size |D|. This output can be interpreted as a probability vector over the dictionary of orientations, D. Ideally, the desired output should be Dirac deltas over the orientations, with intensities representing the volume fractions.

Observe in Figure 1 how, even though we consider a neighborhood as input for the model, the number of layers is manageable: there are only three layers! This is because the first two layers, albeit dense, are shared by the small neighborhoods of the 3D image, reducing the complexity of the model. Moreover, we can extend the same architecture to larger neighborhoods for images with smaller voxels by adjusting the stride and the number of layers. Table 2 exemplifies the various configurations that can be arranged. For instance, if we consider a neighborhood of size 5 × 5 × 5, we can use a first layer with a stride of 1, a second layer with a stride of 2, and a third layer with a stride of 1 outputting 4 × 4 × 4, 2 × 2 × 2, and 1 × 1 × 1 neighborhood representations, respectively.

**Table 2:** Possible configurations of our architecture.

**Neighborhood**	**1st layer**		**2nd layer**		**3rd layer**	
**Size**	**Width**	**Stride**	**Width**	**Stride**	**Width**	**Stride**
3 × 3 × 3	3	1	1	1	1	1
3 × 3 × 3	2	1	2	1	1	1
5 × 5 × 5	4	1	2	1	1	1
5 × 5 × 5	3	2	2	1	1	1
5 × 5 × 5	3	1	2	1	2	1
5 × 5 × 5	2	1	2	2	2	1

The configuration we chose for our experiments is emphasized in gray.

Note that the first two options in Table 2 correspond to known models; the first is an MLP architecture with two hidden layers, and the second configuration is the proposed architecture. By recycling the same perceptron to process all the small neighborhoods, we could reduce the network's complexity compared to AxonNet (Ehrlich and Rivera, 2021). In addition, when processing a complete DW image, the first layer can independently process the small neighborhoods of size two, allowing each to be processed in parallel.

### 3.3 Synthetic data generation

An important part of our model is generating the training data. Given that medical images lack ground truths, our neural network is trained using only synthetic data, conforming our model to a self-supervised method. According to our results, auto-generated data are sufficient for the correct model generalization. Although there are many complex modes for representing a DW signal, for the synthetic data generation, we used a Gaussian diffusion model for being simple and computationally efficient. Therefore, the synthetic data generation consists of defining the variables for the diffusion multi-tensor model, simulating the signals using the DMT model, and generating the representation of the variables to predict. We generate realistic training and validation datasets according to the acquisition protocol of the real DW–signals to be analyzed. Our procedure is described in the following steps.

#### 3.3.1 Fiber representation

We randomly set the orientations of three synthetic fibers for the central voxel; this is done by taking three points uniformly over the unit sphere. Then, vectors whose angular distance is less than min_deg are considered a single fiber. Thus, the number of fibers can be less than three. For all datasets, we set min_deg to 20 degrees.

To set the volume fractions of the previously generated fiber orientations, we follow these steps:

1. We draw two numbers from a uniform distribution $u_{1},u_{2}\sim U\left[0.1,0.9\right]$.
2. We denote the volume fractions corresponding to each of the three fibers, *f*_1_, *f*_2_, *f*_3_, and they are defined as follows:
  *f*_1_ = min(*u*_1_, *u*_2_)
  *f*_2_ = |*u*_1_−*u*_2_|
  *f*_3_ = 1−*f*_1_−*f*_2_
3. We set to 0 the volume fractions corresponding to non-existing fibers, due to the min_deg constrain, and renormalize the values so that *f*_1_+*f*_2_+*f*_3_ = 1.

This way, we can generate voxels containing up to three fibers. Because of step three, ~68% of our dataset contained three fibers, 30% contained two fibers, and a few contained only one fiber. We tried other configurations of these proportions and found no evidence of improvement in the results. We just observed that to predict the three-fiber scenario correctly, the network needed more than half of the data corresponding to that case, given that this scenario seems more challenging. Therefore, we decided to keep these percentages.

#### 3.3.2 Neighborhood generation

We build neighborhoods that diverge slightly from the previously generated central voxels to complete the training and validation datasets. For such a purpose, we do the following:

- We add random perturbations to the vectors generated for the central voxel to form eight different perturbated copies, one for each corner of the 3 × 3 × 3 neighborhood. The perturbations are made by adding small values to the Euler angles defining the tensors, sampled from N(0,0.25)
- We set the fibers of the rest of the neighboring voxels using trilinear interpolation.
- The volume fractions are not modified for any of the neighboring voxels.

**Figure 2:**
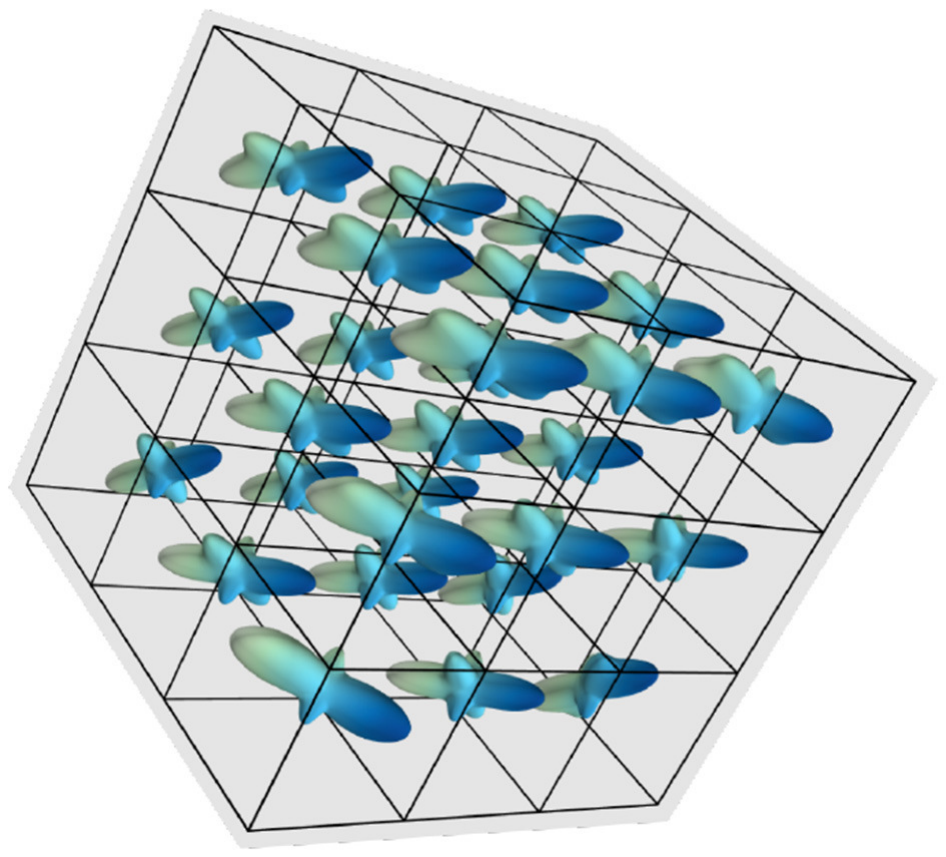
fODF depiction of a datum in the training data, corresponding to a neighborhood of voxels spatially correlated.

Under this procedure, a neighborhood of 3 × 3 × 3 voxels. The corresponding ODF of one datum is shown in Figure 2, where we observe the directions of the three fibers in the 27 voxels of the neighborhood.

#### 3.3.3 Signal simulation

The signals are generated using a DMT model with a tensor eigenvalue calibrated from the *corpus callosum* of an actual DW image (the one used in the qualitative analysis), using the same acquisition protocol G as the real DW signals to be analyzed. This is the bottleneck of the proposed self-supervised approach because of the time it consumes. Fortunately, this generation only needs to be done once for each protocol.

As the image intensity in magnetic resonance images in the presence of noise is shown to be governed by a Rician distribution (Papoulis, 1984; Gudbjartsson and Patz, 1995), we add random Rician noise to the signals of our training and evaluation sets. The noise added is controlled by the signal-to-noise ratio (SNR). First, we randomly choose an SNR into the interval [15, 35] for every datum. With this variation in the noise, we expect the model to generalize well for different DW images. We admit that the choice of this model biases the signals generated using the DMT model. Although these effects are especially noted in the case of neural networks (NN), they are not particular to them. We can say that they are general: Models such as CSD or NNLS generally use the response of the prototype voxel in the corpus callosum (adjusting a mono-tensor) as an element to build the (discrete) signal dictionary. We have tried to ensure this training database is large enough to mitigate this bias. Still, we accept this limitation because the model is designed to be trained with synthetic data due to the scarcity of ground truths in medical images. Nevertheless, as demonstrated in previous studies, we believe the approach can generalize well to actual data (Ehrlich and Rivera, 2021; Karimi et al., 2021).

### 3.4 Ground truth labels

We already mentioned that the desired output is a vector of responses to a dictionary of orientations D of size *d*. To define the ground truth labels, we must define D. The dictionary contains vectors indicating orientations. We set all vectors in the upper hemisphere (positive third dimension) for convenience. To cover most orientations, the vectors are as equally distributed as possible (Jones et al., 1999). An ideal dictionary should have as many orientations as possible to minimize errors, but with 362, we get an excellent level of precision. Now, we define the representation we will use for the output data. The output representation must encode the multiple fibers of the central voxel and their volume fractions. We interpret the output of the model as the volume fractions distributed in the *d* orientations given in the dictionary D. Therefore, as in many deep learning applications, the ground truths, called here *labels*, should reflect that. To that aim, the labels of the central voxel are crafted using a two-step procedure.

In the first step, we compute the *Nearest Element (NE)* of the dictionary for every fiber orientation in the central voxel; that is, we compute the element in D that has the smallest angle to the vector representing the fiber orientation. After computing these labels, we scale them by their volume fractions. Formally, for an orientation *v*, the Nearest Element label of *v* is defined as


$$\begin{array}{l} L_{NE}\left(v\right)=f_{v}\arg\underset{\text{d}\in D}{\min}\arccos\left(\left|v^{⊤}\text{d}\right|\right), \end{array}$$

where *f*_*v*_ is its corresponding volume fraction.

**Figure 3:**
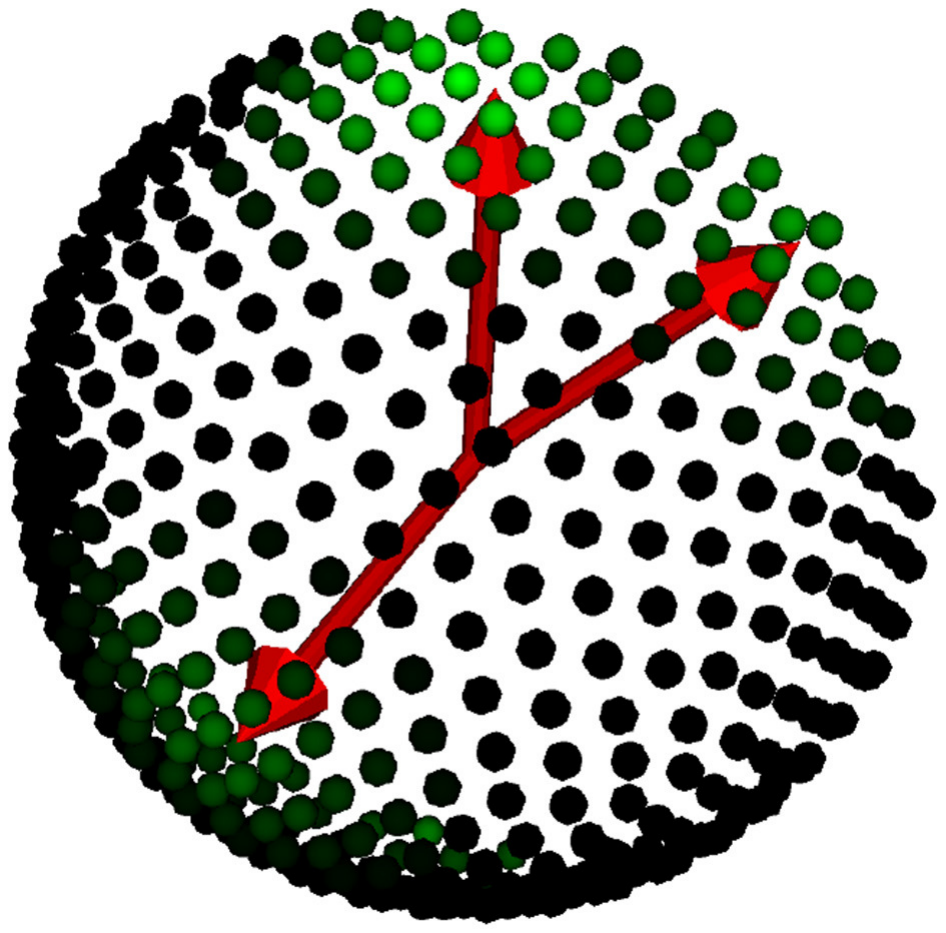
Watson label representation over the dictionary. We display the true orientations in red, while the Watson labels are shown as green intensities over the dictionary of orientations in the hemisphere.

The nearest element labels are our desired output, but choosing this representation introduces a 0–1 loss in training a neural network: Either the model's predicted orientations are right or wrong. However, not all orientations are necessarily wrong since good approximations of the orientations are more desirable than others with a larger angular distance. Therefore, we introduce a more sophisticated representation that encodes a confusion matrix on the orientations. This representation reduces the penalty for small orientation errors. We refer to these labels as *Watson Labels* as they are constructed by adjusting a Watson distribution discretized by the orientations in the dictionary with center on the Nearest Element labels. More formally, we build these labels under the following formula:


$$\begin{array}{l} L_{W\sigma }=W_{\sigma }L_{NE}; \end{array}$$

where $W_{\sigma }\in {\mathbb{R}}^{m\times m}$ is the confusion matrix containing the weights of Gaussian blurring on the sphere with variance σ. Given two directions in the dictionary, ${\tilde{d}}_{i},{\tilde{d}}_{j}$, such weights are defined as $W_{i,j}^{\sigma }=\frac{w\left(d_{\theta }\left({\tilde{d}}_{i},{\tilde{d}}_{j}\right)\right)}{w\left(0\right)}$ for $w\left(a\right)=\frac{1}{\sigma \sqrt{2\pi }}e^{-{\left(\frac{a}{\sigma \sqrt{2}}\right)}^{2}}$, the evaluation of the angles between dictionary directions on a Watson density distribution. *L*_*Wσ*_ are shown graphically in Figure 3. Note that if a voxel contains just one tensor with direction **d**, this construction will do the labels equal to their evaluation on a Watson distribution with mean **d** and variance σ. For more than one fiber, the sphere displays a mixture of Watson distributions with the center in the NE labels, pondered by the volume fractions. Formally, given the Watson blurring of three orientations, $L_{W\sigma }^{1},L_{W\sigma }^{2},L_{W\sigma }^{3}$, the final labels are defined by the following mixture of Watson distributions:


$$\begin{array}{l} L_{W\sigma }=\overset{3}{\underset{i=1}{\sum }}L_{W\sigma }^{i}\;\text{for }i=1,2,3. \end{array}$$

For convenience, σ is expressed in degrees but converted to radians for the computations. In our experiments, we noticed that small values are difficult to train and generate a greater angular error, while a large σ produces less quality in the volume fractions estimations. In this study, values of 8° and 10° are used to construct the Watson labels, *L*_*W*8_ and *L*_*W*10_, respectively.

In case of analysis of ultra-high field data, our method accepts that training data and diffusion labels can be generated with non-Gaussian diffusion models, as the revised by Gallichan (2018), with a slightly additional computational cost.

### 3.5 Training

We train the network with the mean squared error (MSE) loss function between the output tensor and the ground truth labels. This error for two vectors *x, y* ∈ ℝ^*m*^ is defined as


$$MSE\left(x,y\right)=\frac{1}{m}∥x-y{∥}^{2},$$

where *x* are the normalized signals. MSE is the loss function used per excellence for regression problems when training neural networks. This is due to the equivalence of minimizing the quadratic norm to the maximum likelihood estimator by assuming a Gaussian distribution for the noise. Although many specific loss functions exist for different tasks nowadays, MSE typically exhibits good training accomplishments. This study is not the exception; we use MSE as a loss function to train our models.

The optimizer used for training was Adam, with a learning rate of 0.002. We decreased this learning rate by a factor of 0.2 when reaching a plateau in the training loss. The training was stopped when the loss, computed in an independent validation set, did not decrease for 10 epochs. No regularization was used. The training set consisted of 20,000 examples, and the validation set consisted of 5,000 examples of 27 voxels generated with the procedure introduced in the previous section. The number of signals depends on the acquisition protocol of the evaluation's datasets.

### 3.6 Experimental methodology

The experiments were focused on determining the quality of the inferences produced by our model. To that end, we tested how well it can infer three crucial elements of the intra-voxel structure: the number of fibers, their orientations, and the volume fractions. For this purpose, we conducted some experiments and used several metrics. We describe the experimental setup in this section.

Our experiments are divided into two parts according to the objectives pursued. The first set of experiments tested how the different hyperparameters affected the model's prediction. These values are the number of neurons in each layer, *n*_1_ and *n*_2_, and the parameter σ, the variance of the Watson distributions used for the labels. We also validated the model by comparing its performance with classical MLPs. When choosing the size of the layers, we were interested in a model with a low computational complexity without compromising good performance in the precision of the inference. For the validation of the model, our reference is the Multi-Layer Perceptron *AxonNet* (Ehrlich and Rivera, 2021), as a previous study suggested a good performance. To that aim, we consider the predictions of the two MLPs:

- A MLP consisting of seven linear layers with neurons ranging from 512 to 4,096. This model takes the voxel's neighborhood as input, just as our proposed model. For the rest of this study, we refer to this model as *Neighborhood-MLP*.
- A MLP that evaluates the signals of the central voxel, ignoring the neighboring voxels. This slightly smaller model has six dense layers and a range of neurons between 512 and 2048. We refer to this model as the *Voxel-MLP*.

We refer to Ehrlich et al. in 2020 preprint (Ehrlich and Rivera, 2021) for more detailed information about these models.

The second set of experiments was conducted to test the performance of our model in a well-known phantom image and compare our estimations with the estimations produced by CSD. To that end, we evaluated both models using simulated data given as evaluation on the ISBI 2013 HARDI reconstruction challenge (ISBI, 2013). The signals of this phantom image were simulated through a more complex procedure that differs from the one used for the training data. The data consist of images of size 50 × 50 × 50, and it is available in two acquisition protocols: a DTI scheme with 32 signals and *b*-values of 1200 *s*/*mm*^2^, and a HARDI scheme with 64 directions and *b*-values of 3000 *s*/*mm*^2^. We concatenate both images for our experiments, producing a multi-shell protocol of 96 signals. As the ground truth fixels are given for each voxel, we can evaluate the quality of the predictions. The quantitative evaluation consists of several metrics over the fODFs estimated by each model and the time each model takes to produce the estimations. One crucial element for the comparison is the metrics used to evaluate the models. We dedicate the following section to introduce those metrics.

Finally, our experiments are completed with a visual inspection of the estimations on data from a real healthy male subject from the Stanford HARDI dataset (Rokem et al., 2015). The set consists of single-shell data with *b*-values of 0 and 200 *s*/*mm*^2^, with a protocol of 160 gradient directions. In this inspection, we focused on evaluating the predictions on areas with multiple crossing fibers and some common mistakes in the estimations found in the literature.

### 3.7 Performance metrics

We can assess several aspects in measuring the quality of the inferred microstructure. For instance, Canales-Rodŕıguez et al. (2019) compiled five types of error that can be taken into consideration to evaluate the precision of the estimated peaks: the angular error, the volume fraction error, the number of fibers over-estimated, the number of fibers under-estimated, and the success rate. A fiber peak is an estimated fiber orientation chosen from an orientation distribution function and associated with a volume fraction. To evaluate the precision in fiber orientations for a datum, they propose the *angular error* defined as


$$\begin{array}{l} \theta_{e}=\frac{1}{M_{true}}\overset{M_{true}}{\underset{k=1}{\sum }}\underset{m}{\min}\left\{\arccos\left(\left|e_{m}^{⊤}v_{k}\right|\right)\right\}, \end{array}$$

where *M*_*true*_ is the true number of fiber populations; *e*_*m*_ is the unitary vector along the *m*-th detected fiber peak, and *v*_*k*_ is the unitary vector along the *k*-th true fiber orientation.

In addition, Canales-Rodŕıguez et al. (2019) proposed using the mean absolute error as a *volume fraction error*:


$$\begin{array}{l} \Delta f=\frac{1}{M_{true}}\overset{M_{true}}{\underset{k=1}{\sum }}|f_{m}-f_{k}|. \end{array}$$

**Figure 4:**
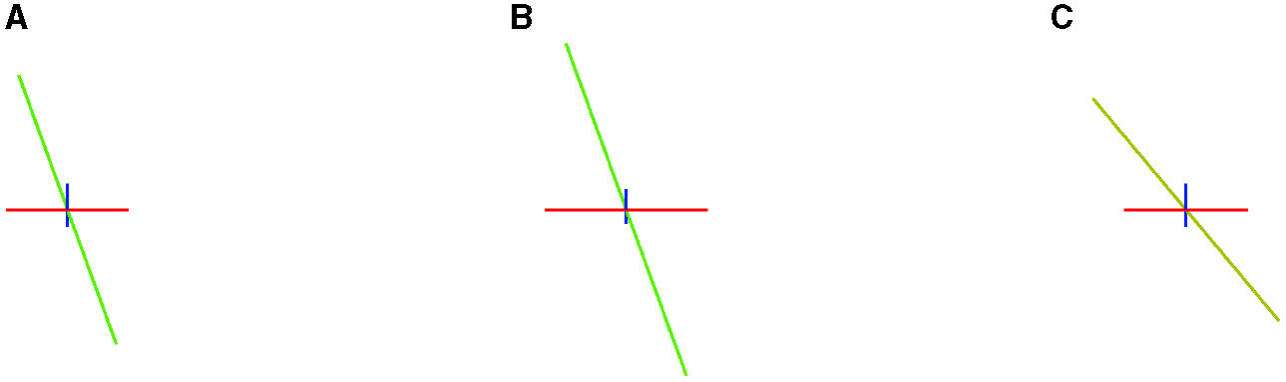
Example of how small differences in estimation can produce an unsuccessful estimation. In the left image **(A)**, three vectors of volume fractions of 0.5 (blue), 0.35 (green), and 0.15 (red). In the center **(B)**, the same vectors but with volume fractions of 0.4, 0.41, and 0.19, a different relative order of predominance. In the right-hand image **(C)**, the same volume fractions but a vector with a distance of 20.1° w.r.t. the first image.

To evaluate how well a model estimates the number of fibers, Canales-Rodŕıguez et al. (2019) propose computing the mean number of fibers over-estimated per voxel, *n*^+^, and the mean number of fibers under-estimated, denoted by *n*^−^. In addition, the cited study defines the *Success Rate*, *SR*, as the proportion of voxels in which the algorithm estimates the right number of fiber compartments, calculated with an angular error inferior to a given value (25° in our experiments), and the correct relative order of volume fraction among the predicted fibers. The Success Rate is a metric that indicates the accomplishments in the estimation. In Figure 4, we observe how small variations in the solution produce unsuccessful estimations.

Canales-Rodŕıguez et al. (2019) propose a metric that considers the errors mentioned above to facilitate the comparisons between different methods. The global relative performance (GRP) of a method *i* is defined as


(1) $$\begin{array}{l} GRP\left(i\right)=\frac{\theta_{i}}{\langle \theta \rangle }+\frac{\Delta f_{i}}{\langle \Delta f\rangle }+\frac{n_{i}^{+}}{\langle n^{+}\rangle }+\frac{n_{i}^{-}}{\langle n^{-}\rangle }+\frac{1-SR_{i}}{1-\langle SR\rangle }, \end{array}$$

where 〈χ〉 denotes the mean value of metric χ for all methods in the comparison.

The global relative performance is helpful as it summarizes the performance in one number, facilitating comparisons between methods. However, one disadvantage of this formula is that it equally weights the five types of errors. Moreover, any specific weighting choice can be controversial because such metrics cannot be directly compared. Furthermore, each type of error is normalized by the mean error, so for the two methods, a mean overestimation between 0.01 and 0.001 costs the same as the overestimation between 1.0 and 0.1, even though the second difference is more relevant. Because of this disadvantage, we still need to compare each metric individually. As an alternative, we evaluate using the Earth Mover's Distance to compare methods. The main EMD advantage over the metric (1) is its interpretability: EMD is the least amount of energy necessary to transform one collection of items into another.

The *Earth Mover's Distance (EMD)*, also known as the 1-Wasserstein distance, defines a distance between histograms and probability measures (Peyré et al., 2019). Intuitively, given two distributions, one can be seen as a mound of earth spread in space, the other as a collection of holes in the same space. Then, the EMD measures the least amount of work needed to fill the holes with dirt. A formal definition of the Earth mover's distance over a general metric is as follows (Andoni et al., 2008): Consider a metric space *X* endowed with distance function *d*_*X*_. For two sets *A, B*⊂*X* of size *n*, its cost matrix $C\in {\mathbb{R}}_{+}^{n\times n}$ is defined as *C*_*i, j*_ = *d*_*X*_(*A*_*i*_, *B*_*j*_) for elements *i* and *j* in *A* and *B*, respectively. Given two probability vectors **a**, **b** ∈ ℝ^*n*^ that provide weights to the elements of *A* and *B*, respectively, the Earth Mover's Distance (EMD) between **a** and **b** is defined as


(2) $$\begin{array}{l} \underset{P\in {\mathbb{R}}_{+}^{n\times n}}{\min}\underset{i,j}{\sum }C_{i,j}P_{i,j}\text{ subject to }PI=\text{a},\;P^{⊤}I=\text{b}. \end{array}$$

EMD is proved to be a distance satisfying symmetry and triangle inequality, and it also naturally extends the notion of a distance between elements to that of a distance between sets or distributions of elements. One of the crucial properties of the EMD is that it is a weak distance; that is, it can be used to compare singular distributions whose supports do not overlap and to quantify spatial shifts between the support of two distributions. This sets EMD apart from other notions of distance. The Kullback-Leibler Divergence, for instance, requires overlapping distributions to be useful (absolute continuity is needed) (Peyré et al., 2019). Moreover, other classical distances are not even defined between discrete distributions. For example, the L2-norm can only be applied to continuous measures with a density concerning a base measure, and the discrete L2-norm requires that positions (*x*_*i*_, *y*_*j*_) take values in a predetermined discrete set to work correctly.

The properties of EMD make it suitable for our needs. We can compare two distributions over the sphere as two fODFs. In other cases, we can compute the EMD between smooth distributions and isolated peaks; for example, we can compare Dirac deltas, which indicate the actual fiber orientations, and a fODF estimated by a method. The EMD also differs when representing distributions with different modes, which is useful when multiple fibers exist. For example, when comparing two results with a different number of detected fibers, EMD auto-assigns the orientation predictions to the closer one by minimizing the work needed to move one into the other, thus eliminating the need for manual pairing. Moreover, EMD considers the variance between distributions, which is useful when comparing certainty among the estimated orientations. To get intuition on how the EMD works, see the two special cases provided in the Supplementary material.

## 4 Experiments and results

In the first experiment, we evaluated the performance of our neural network with a different number of neurons in each layer, denoted by *n*_1_ and *n*_2_ for layers 1 and 2, respectively. In general, all models accomplished good results, from the smallest model with 128 neurons in the first layer and 256 in the second to the largest model consisting of 1,024 and 1,536 on each layer. Generally, performance improves proportionally to the model's size, so the largest models produce better results (Supplementary Table 1). However, the performances were barely different compared to the drawbacks of increasing the size (Supplementary Table 2). For example, the model with 256 neurons in the first layer and 512 in the second layer has an MSE over an evaluation set of 7.01*e*−06, and the model with 768 neurons in the first layer and 1024 in the second layer has an MSE of 6.98*e*−06. Although there is a slight difference in the results, the number of parameters is five times larger in the second model. After this first evaluation, even though the larger models seemed better, we chose a manageable model that could be executed on a CPU in a reasonable time. Thus, from now on, the results presented here correspond to the model with 2.54 million parameters and a size of 512 in the first layer and 512 in the second. Henceforth, we refer to this specific neural network, trained over labels of σ = 10 as the Local Neighborhood Neural Network *W*10 (LNNN-W10) and the one trained with a variance of 8 as the Local Neighborhood Neural Network *W*8 (LNNN-W8). When omitted the last part, as in *LNNN*, we refer to the *W*10 model.

We validate the Local Neighborhood Neural Network by comparing its performance with the multi-layer perceptrons mentioned in Section 1. Table 3 shows the results obtained by those models, all trained with the same training data previously described. From this table, we can make two observations. The first one is that considering a neighborhood instead of one voxel definitively improves the results as the Neighborhood-MLP and the LNNN obtained better results than the Voxel-MLP. The second is that a multi-layer perceptron capable of achieving the level of performance of the LNNN needs 19 million parameters. Thus, LNNN has less than 15% of parameters. In addition, our proposal can lower memory complexity, reduce the number of parameters, reduce computational cost, and lower the training computational time. The training of our model was performed in 531 s on an 8-core AMD CPU processor @ 4.5 GHz. This time is relatively small for using only CPUs. That time is well under a typical adquisition duration once the protocol is fixed, which facilitates the use of our model *in situ*. For example, once the protocol is fixed, this protocol information can be sent to a server, and the acquisitions can be sent to the server for processing after the acquisition is finished, getting the results in no time.

**Table 3:** Comparative between the AxonNet (Ehrlich and Rivera, 2021) and the proposed models.

**Model**	**Parameters (1e6)**	**Training time**	**Eval. MSE**	**Eval. MAE**
Voxel MLP	4.25	465 s	1.44e-5	2.06e-3
Neighborhood MLP	19.59	1,733 s	1.15e-5	1.89e-3
**LNNN-W10**	**2.54**	**531.7 s**	**6.96e-06**	**1.76e-3**

We highlight the smallest values in bold.

**Figure 5:**
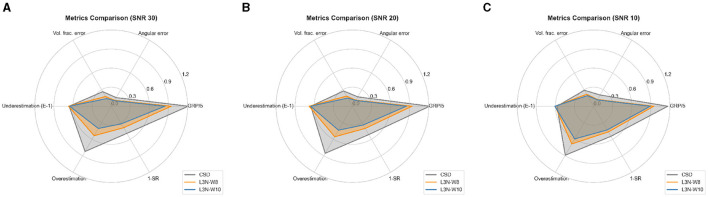
Metrics on ISBI 2013 dataset with an SNR of 30 **(A)**, 20 **(B)**, and 10 **(C)**. Note that underestimation is scaled, GRP is divided by the number of metrics, and the failure rate (1 − *SR*) is given as a ratio between 0 and 1.

In the second set of experiments, we observed that our models' performance is competitive with CSDs. We used the ISBI 2013 reconstruction challenge dataset to compare our models' performance with CSDs. The first thing to note is the computational times. The CSD method took 3.5 min to process the image, while our models took 9 min, still a manageable time. In Table 4, we report the mean EMD obtained by computing the distance between the estimated fODFs and the true fixels. At least one of the networks obtained a lower mean EMD over the ISBI 2013 test dataset for data with SNR of 30 and 20. We highlight the results obtained by the LNNN in the image with a low signal-to-noise ratio. The training dataset contained only voxels with an SNR as low as 15, and the model seems to perform well on the image with an SNR of 10. We also computed the errors per type. Figure 5 depicts a graphical summary of the proposed LNNN variants (W8 and W10). The GRP for the image with a SNR of 30 was 4.24 for LNNN-W10, 4.69 for LNNN-W8, and 6.07 for CSD. Similar magnitudes were obtained for noisier images.

**Table 4:** Comparative of CSD and LNNN.

**Method**	**SNR 30**	**SNR 20**	**SNR 10**
CSD	0.4109	0.4227	*0.4371*
**LNNN-W10**	*0.4045*	0.4251	0.4444
**LNNN-W8**	0.4169	*0.4135*	0.4536

Mean EMD using phantom images with different noise levels. We highlight the lowest value per noise level in italics.

Table 5 presents a performance comparison using the metrics compiled by Canales-Rodŕıguez et al. (2019). We found it relevant to characterize the apparent advantage of CSD over LNNN in underestimation by observing what happens to the *SR* on voxels with the presence of multiple fibers. When multiple fibers cross, it is important to recognize the correct number of fibers and not miss an axonal bundle. Table 5 summarizes how the five metrics behave in voxels with three fibers. In this particular case, our model has a clear advantage over CSD in all image metrics, especially in the *SR*.

**Table 5:** Angular error, volume fraction error, overestimation, underestimation, and failure rate of CSD and LNNN for voxels with the presence of three fibers; lower is better.

**SNR**	**Method**	** ${\bar{\theta }}_{e}$ **	** $\bar{\Delta f}$ **	** ${\bar{n}}^{-}$ **	** ${\bar{n}}^{+}$ **	**100−*SR* (%)**	**GRP**
	CSD	16.9	0.304	1.081	0	82.3%	4.11
30	LNNN	15.6	0.2695	0.9952	0	80.86%	3.83
	CSD	16.8	0.2866	1.048	0	85.17%	4.09
20	LNNN	15.6	0.2695	0.9952	0	82.30%	3.78
	CSD	19.9	0.2824	0.9522	0	86.12%	4.12
10	LNNN	16.6	0.2671	0.9474	0	79.90%	3.88

**Figure 6:**
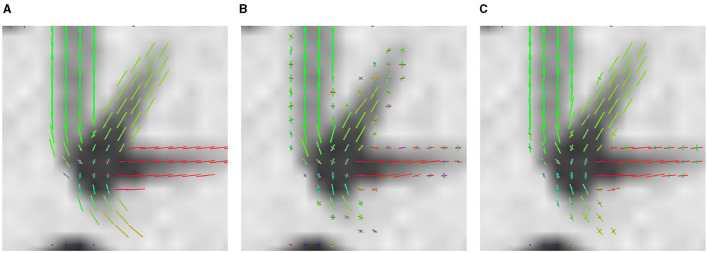
Results on ISBI 2013 2-shell Phantom image. Axial view of the fixels in a crossing region. **(A)** GT fixels. **(B)** CSD fixels. **(C)** LNNN-W10 fixels.

**Figure 7:**
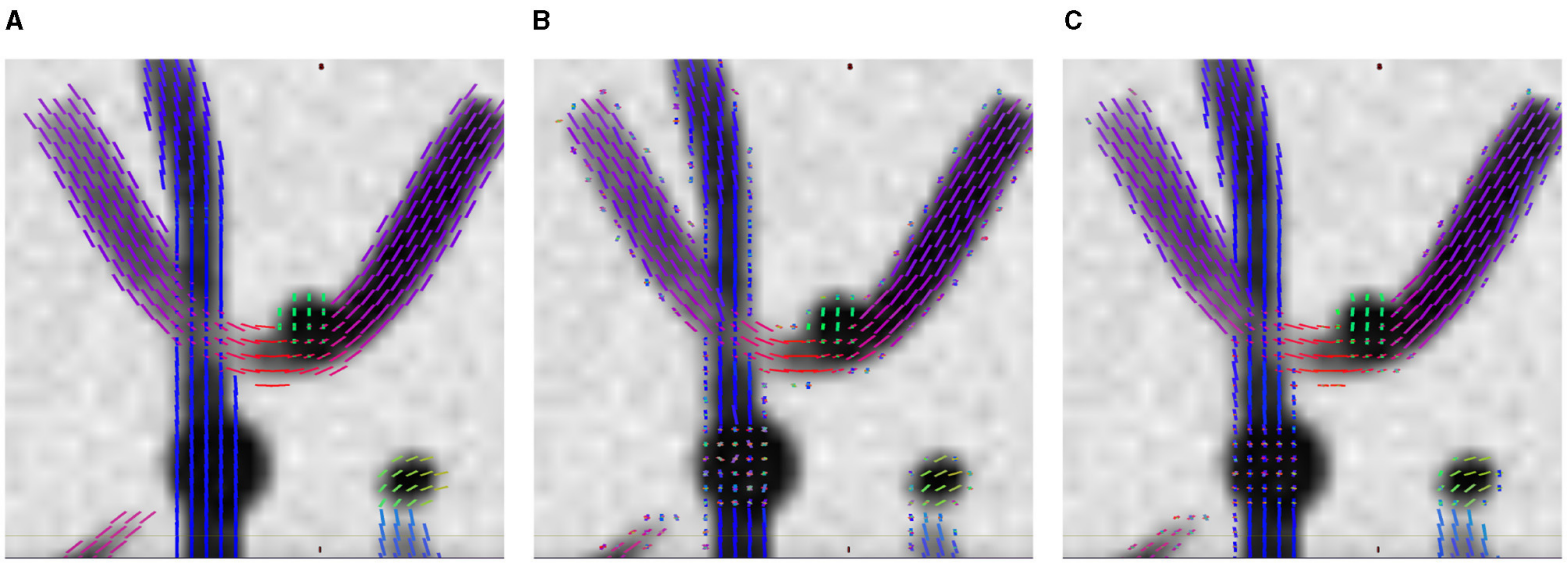
Results on ISBI 2013 2-shell Phantom image. Axial overview of the fixels. **(A)** GT fixels. **(B)** CSD fixels. **(C)** L3N-W10 fixels.

**Figure 8:**
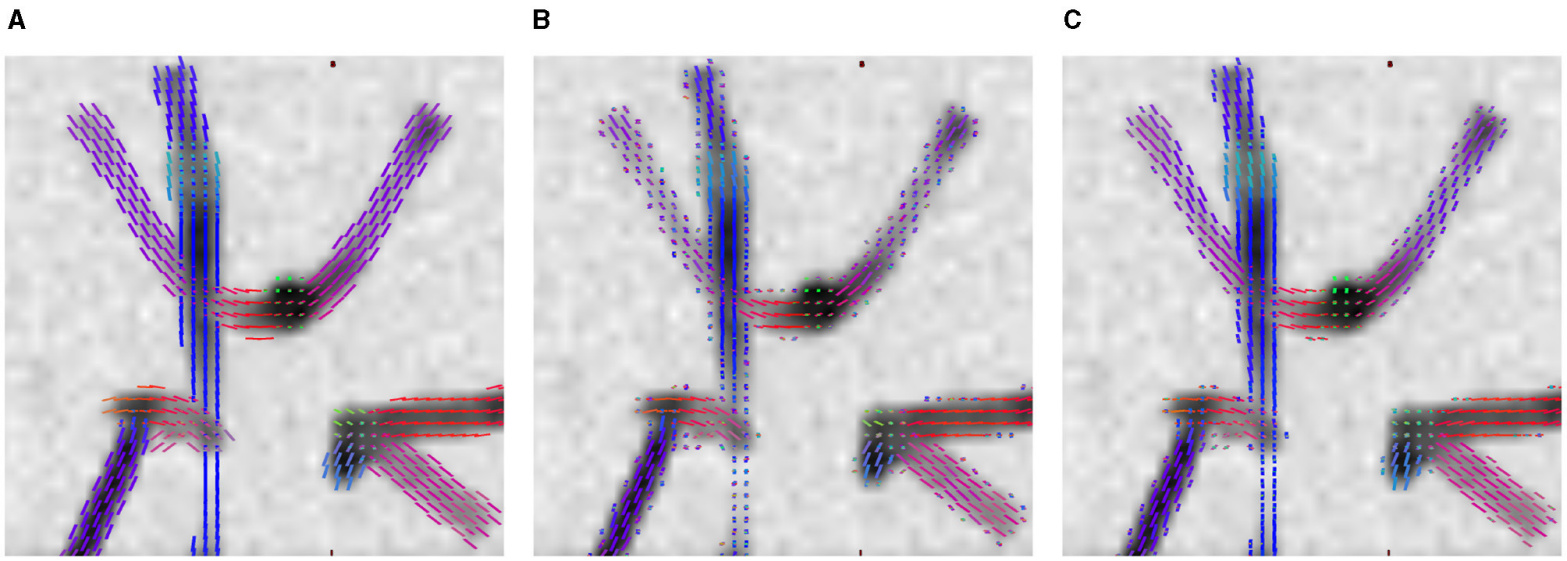
Results on ISBI 2013 2-shell Phantom image. Axial overview of the fixels. **(A)** GT fixels. **(B)** CSD fixels. **(C)** L3N-W10 fixels.

The advantage of LNNN over CSD can be visualized on the fixels. In Figure 6, we plot the fixels of both methods for a fiber crossing. Observe how noisy the CSD solution is. This problem is consistent for several slides. For example, in Figure 7, we have a circular area where the fibers cross a volume with free diffusion. The CSD's estimated fixels in this area miss the blue bundle in various voxels. At the same time, even though it also introduces spurious orientations, our model manages to estimate the blue bundle in most of the voxels correctly. Finally, observe in Figure 8 how our model correctly estimates the number of fixels while CSD introduces several more. This represents a problem for the tractography as the tracking algorithm may follow non-existing fixels.

**Figure 9:**
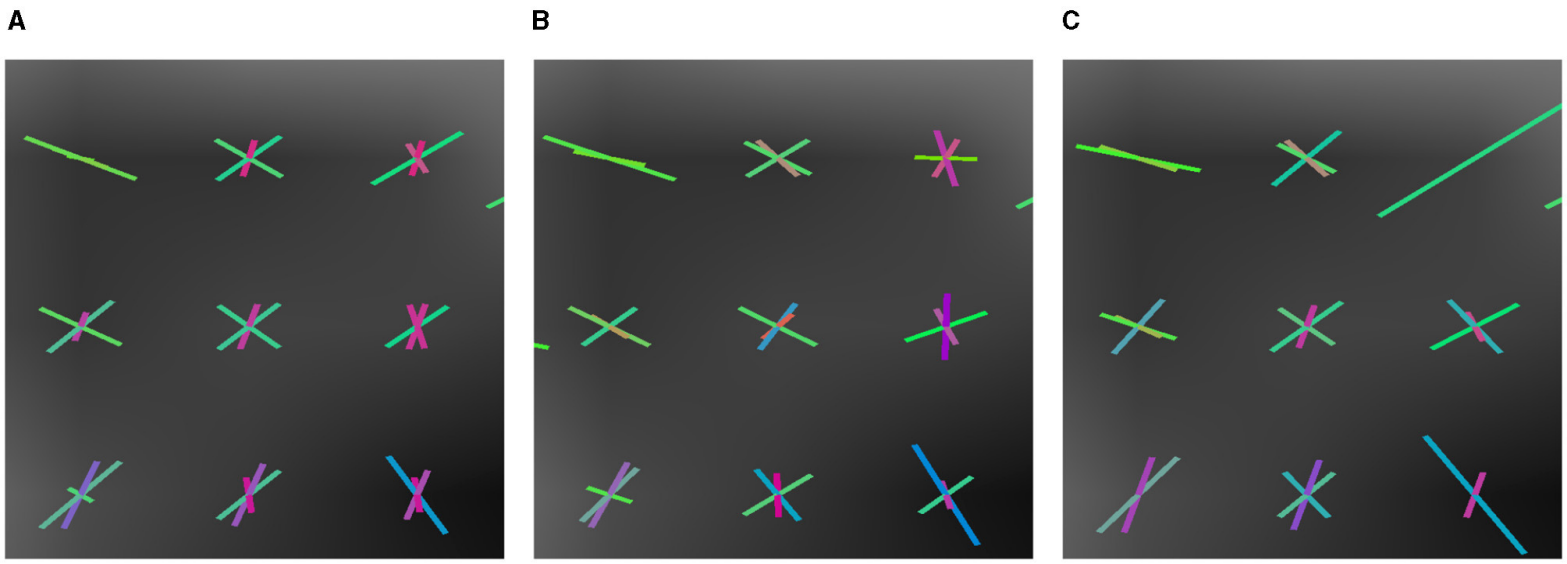
Results on ISBI 2013 2-shell Phantom image. Sagittal zoom-in of the fixels in a crossing region. **(A)** GT fixels. **(B)** CSD fixels. **(C)** LNNN-W10 fixels.

We also illustrate various estimations in Figures 6, 9 that can produce the difference in their success rate. We note how, in some voxels, CSD tends to add spurious fixels or estimates with the wrong orientations. Compare the central fixels of Figures 9A–C with the explanation of the *SR* in Figure 4.

We also evaluated the quality of our method in authentic DW images. For such a purpose, we use the Stanford HARDI image (Rokem et al., 2015), consisting of a single-shell protocol of 160 signals. We compared the quality of the micro-structure recovered using LNNN and CSD. The procedure to extract the fixels consisted of two steps. First, we recovered the fODF using each method; in the case of LNNN, we treated the network's output as the fODF. Then, we recovered the fixels via a peaks estimator with each function, limiting to a maximum of three fibers and a peak threshold of 0.2. Finally, we inspect the visualizations of the fixels (scaled by the volume fractions) using MRView from MRTrix. We present the visualization of the more representative differences between the methods.

**Figure 10:**
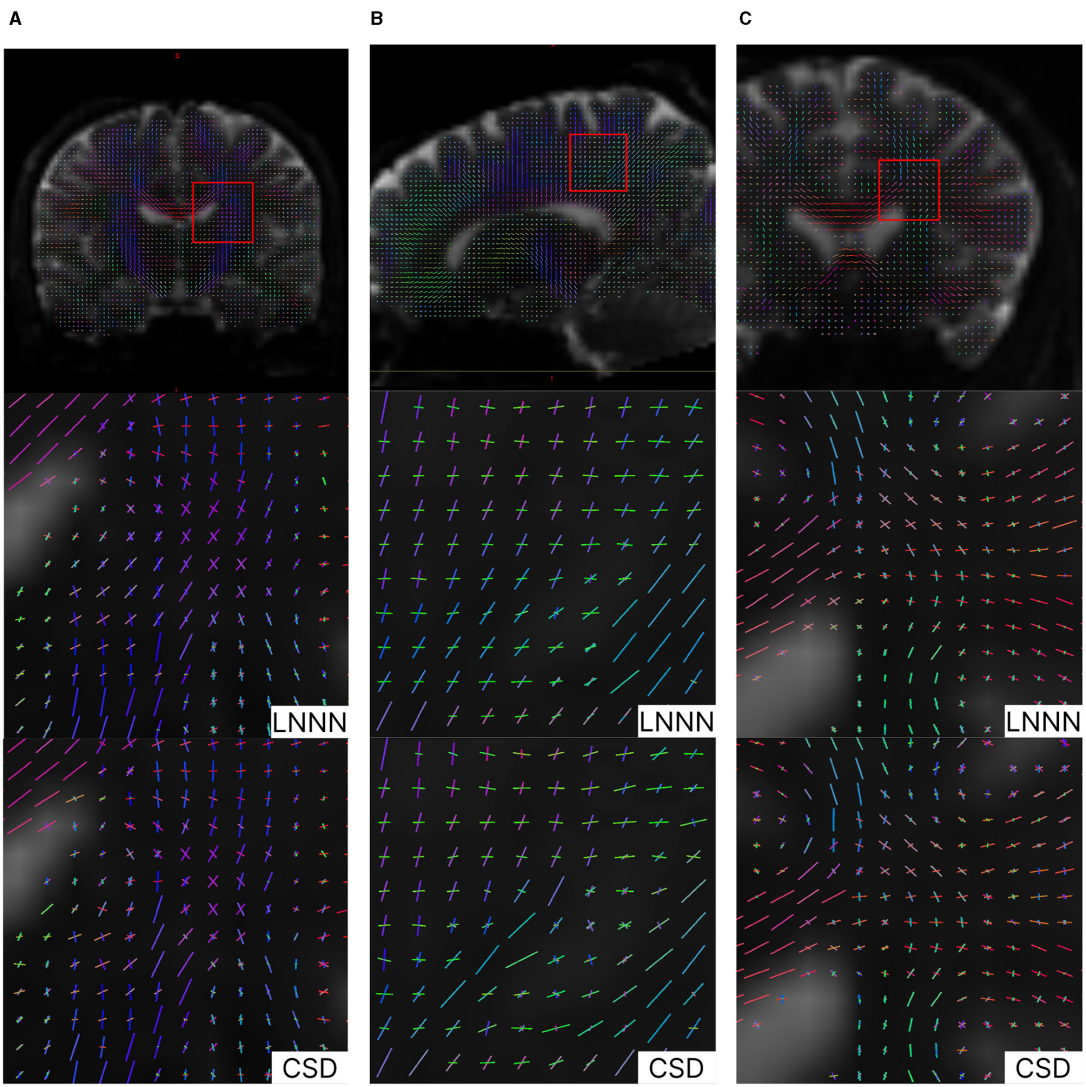
Results on Stanford HARDI image. Upper images illustrate the location analyzed. Note the discontinuity estimated by CSD in the blue fixels in **(A)** and the discontinuity estimated by CSD in the fixels colored in green in **(B)**. In general, we appreciate a slightly better spatial coherence in the estimations of LNNN. **(A)** Coronal view of crossing fibers at the level of the corona radiata. **(B)** Saggital view showing crossings with the corona radiata. **(C)** Coronal view: axons of the CC intersect cortical/thalamic projections.

**Figure 11:**
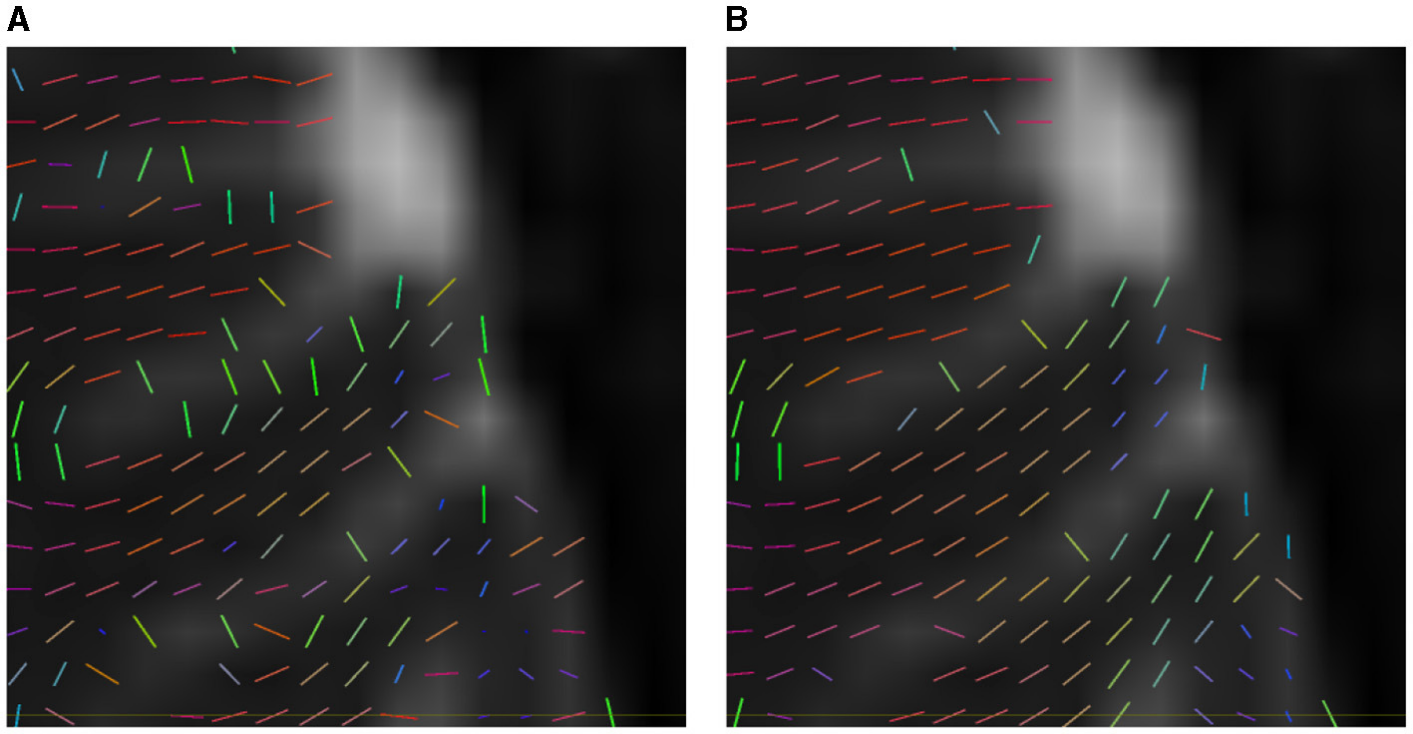
Comparative of estimated prominent fixels on Stanford HARDI image at gyral blades. **(A)** CSD. **(B)** LNNN.

In Figure 10, we plot the fixels recovered by each method, respectively. This overview of the whole slide shows that both methods behave similarly, especially in regions where only one fiber is detected; for instance, one can observe in Figures 10A, C that both methods agree in the estimations of the corpus callosum (mainly in red) and the fibers mainly colored in green. However, there are certain differences that we want to point out. First, where the fixels in blue cross the purple/pink fiber, CSD estimations miss some purple/pink estimations, while LNNN does not. Judging by the orientation of the purple/pink fixels and the voxels where both methods agree, LNNN is more likely to be correct in the estimations. This type of 'interruptions' is common in the estimations obtained using CSD; for example, in Figure 10B, we observe the same behavior with the green and blue fixels. The robustness of our model in this type of situation is probably attributed to the information about the neighborhood it processes. We note that both methods differ the most in the folds near the border. Analyzing the predominant orientation, we note that CSD estimates the fanning better than LNNN in the gyral blades, while LNNN suffers from the effect known as gyral bias (Wu et al., 2020). In Figure 11, we plot the fixel with the greatest volume fraction. In this figure, it can be observed that the fanning is recovered in the estimations of CSD but not in the estimated fixels of LNNN. LNNN recognizes the fixels going in the orientation orthogonal to the frontier, but in the fixels with the second larger volume fraction (see Supplementary Figure 3). In this case, judging by the existing literature (Wu et al., 2020), the estimations of CSD are preferred. That is proposed to be addressed in further study.

## 5 Discussion

**Figure 12:**
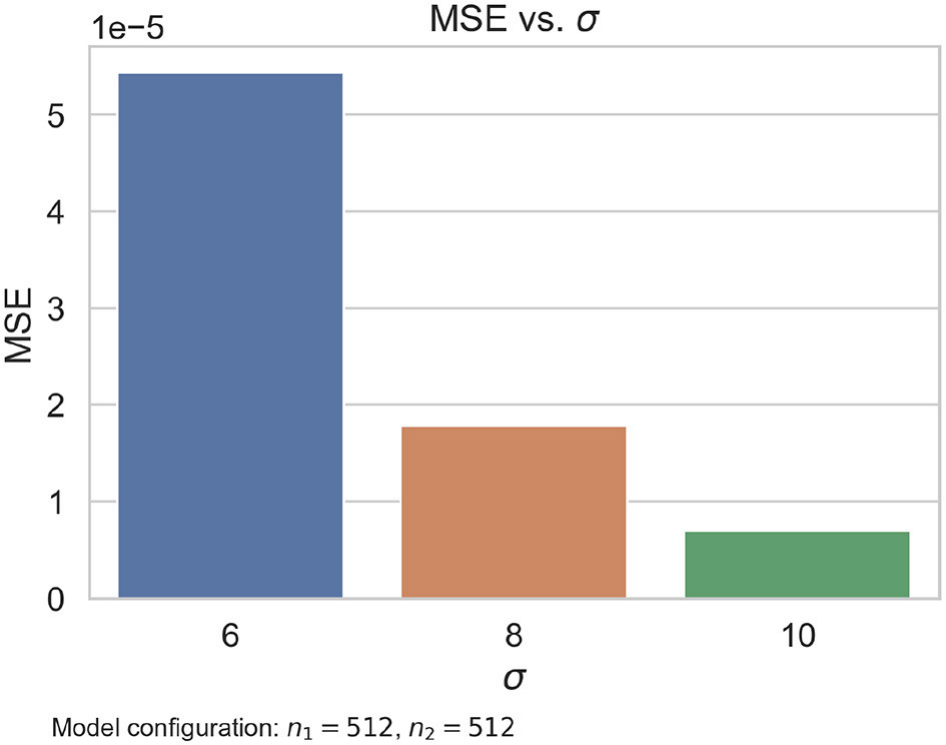
Comparative of the same model configuration trained with Watson labels with a different σ parameter. We note that σ = 10 produces the smallest error.

As was noted in the experiment summarized in Table 3, the number of neurons per layer in the proposed model has a very small effect on the results. What seemed to have a greater effect on the performance is σ, the variance of the Watson distributions used as targets in the training. We thought that the smaller this value was, the sharper the estimated peaks would be. However, the synthetic data evaluation results show that the estimation quality worsens. For example, Figure 12 presents a comparison of the mean squared errors obtained by the same model configuration trained with different variances in the Watson labels. The same gap can be observed for all the configurations we tested. We observed that the variance of the Watson distributions should be large enough to train the model properly.

Analyzing the metrics compiled by Canales-Rodŕıguez et al. (2019), we observe that both variants of the LNNN get results of good quality. Figure 5 resumes the evaluation of CSD and LNNN using these metrics and the GRP (divided by the number of metrics). If we look at this last indicator, our models are better ranked than CSD with a lower GRP. Note that the goal is to recover the intra-voxel structure, so here, we evaluate the accuracy of the fixels rather than the fODF, which was implicitly evaluated by the EMD. As our goal is to describe the fixels, and the Watson distributions were designed for having q stable the training (to have a smooth convergence), it makes more sense to evaluate the metrics in Figure 5. Moreover, computing the EMD is computationally expensive because it involves solving a linear program for each voxel. Consequently, we recommend using the metrics compiled by Canales-Rodŕıguez et al. (2019) for a quick evaluation and use EMD for comparing estimates between the same model, as in this case, the fODF follows the same distribution. Thus, in this case, EMD can be a good metric for comparing the overall score.

In Figure 5, we note that LNNN-W10 has the lower GRP, improving especially in the angular precision and improving evaluations in 4 out of 5 metrics. One important feature to note is that LNNN avoids overestimation when compared with CSD, with a slight increase in the underestimation of fibers. Conversely, CSD has a slightly better underestimation but a much worse overestimation. Thus, we can conclude that LNNN is better at estimating the number of fibers in the voxels. GRP not only measures the estimation of the correct number of fibers but also weights how accurate they were regarding the angular precision, volume fraction accuracy, and the number of rightness defined by the success rate. Let us extend the observations about how to interpret this last metric. As explained before, *SR* can distinguish between spurious and correct fixels. In this case, we observe that LNNN maintains *SR* over 60% in data with SNRs of 30 and 20 and over 50% for data with SNRs of 10. Our results indicate the robustness and reliability of our method, even in the presence of significant image degradation. We also performed qualitative validation on real data, showcasing the applicability of our model in real-world scenarios. The estimates obtained from our model showed greater spatial consistency compared to CSD (see Figure 10). However, it is important to note that CSD estimations are preferred at voxels where our method is susceptible to gyral bias (see Figure 11), ensuring the most accurate results found in the literature in such scenarios. In future study, we plan to explore more complex scenarios within the training data, including neighborhoods with partial volume or fanning gyral blades. By incorporating such complexities, we aim to enhance the versatility and adaptability of our method across a wider range of imaging scenarios. In addition, we will investigate more sophisticated architectures that can operate independently of the specific acquisition protocol, further expanding the versatility and practicality of our approach.

We also explored using the Earth Mover's Distance (EMD) as a metric to compare the precision of estimations between different methods. The EMD has been widely used to measure the dissimilarity between probability distributions. We hypothesized that it could provide a meaningful measure for evaluating the accuracy of our model's estimations. Through experimentation, we found that the EMD provided valuable insight into the precision of the estimates. It captured the differences in spatial distributions between our model and other methods, providing a more nuanced understanding of their performance. However, during our investigation, we encountered a significant limitation that made the EMD less reliable for comparing our model with CSD.

The EMD has a clear advantage over GRP: It does not depend on the results obtained by other methods to be computed and used for comparisons. As the formula states, to calculate the GRP, it is necessary to calculate the required metrics of all the methods in the comparison as the mean errors normalize the values. On the other hand, EMD can be computed for a method without the need for the results of the different methods' results, and the best-ranked method is the one with the lowest EMD value over the same data.

Even though EMD seems to have some advantages over GRP, this metric has two clear disadvantages. The first drawback is the increased computational complexity as evaluating the mean EMD over a dataset with *V* voxels requires solving *V* optimization problems. This significantly prolongs the evaluation time. Another drawback relates to the treatment of variance. When using EMD on distributions, it places importance on variance. However, if our main interest lies in comparing fixels rather than fODFs, the penalizing variance may not be desirable since we are primarily concerned with peak orientations rather than the dispersion of the fODF.

Our proposed model incorporates the variance of fODFs as a crucial parameter. We discovered that by adjusting this parameter, we could manipulate the EMD scores, thus potentially misleading the comparison between our model and CSD. Because of this inherent vulnerability and the computational cost associated with calculating the EMD, we noted that the EMD may not be a trustworthy metric for evaluating the precision of estimates when comparing our model to CSD. Relying solely on the EMD could lead to misinterpretation and misrepresentation of the comparative performance of the two methods.

A design criterion for our model was to develop an efficient and effective method for inferring structural features rather than tissue types, as neurite orientation dispersion and density imaging (Zhang et al., 2012) and other similar models. This imposes a limitation to our model. However, once the structure is determined with our method, the results can be post-processed to estimate tissue compartments. This is left for future study.

## 6 Conclusion

We have introduced a novel method for intra-voxel structure analysis using a neural network. Our method leverages the spatial correlation of voxels within the architecture, enabling efficient inference while minimizing the number of parameters required. This approach exploits the inherent relationships between neighboring voxels, resulting in improved performance when analyzing complex intra-voxel structures or voxels with a high level of noise.

We have developed a method for simulating voxel neighborhoods to address the challenge of acquiring ground truth data. This allows for a self-supervised approach, eliminating the need for ground truth annotations. The training data generated closely mimic real-world scenarios, enabling the training of a model that can be successfully applied to real data. This innovation opens up new possibilities for analyzing intra-voxel structures with deep learning approaches without relying on manual annotations. We conducted comprehensive quantitative validation to evaluate our proposal performance using phantom images that closely resemble real *in vivo* data. Our model exhibited competitive performance against one of the most widely used state-of-the-art methods, demonstrating its effectiveness in accurately analyzing intra-voxel structures. Compared with CSD, our model performed better in 5 out of 6 metrics, particularly in images with high noise levels. These metrics are angular error, accuracy in volume fraction estimation, success rate, overestimation, and general relative performance. Qualitatively, LNNN shows better spatial consistency in analyzing certain areas of real brain images than CSD.

Our method offers some advantages in terms of computational efficiency compared to other deep learning approaches. It has a low computational cost and can be easily parallelized, facilitating fast and scalable implementation. Because of its small size, the network's training can be done even on a CPU in a relatively short time. However, we acknowledge that the simulation of signals for training data poses a computational bottleneck. This can be challenging, especially when the imaging protocol is frequently changed. Further optimization strategies are needed to overcome this limitation and streamline the training process.

In addition to the aforementioned contributions, we investigated the Earth Mover's Distance (EMD) as a comparison metric between analysis methods for estimating intra-voxel structure. However, we concluded that EMD may not be a trustworthy metric for evaluating the precision of estimates when comparing our model to CSD because of the EMD sensibility to the smoothing effect produced by the Watson Labels and its computational cost.

## Data Availability

Publicly available datasets were analyzed in this study. This data can be found here: http://hardi.epfl.ch/static/events/2013_ISBI/testing_data.html, ISBI 2013 Reconstruction Challenge.
