# Supplementary material for: Light-weight neural network for intra-voxel structure analysis
Source: Front Neuroinform. 2024 Sep 9;18:1277050. doi: 10.3389/fninf.2024.1277050 (PMC11417038; doi:10.3389/fninf.2024.1277050)
Supplement: Supplementary file 1 [file Data_Sheet_1.PDF]

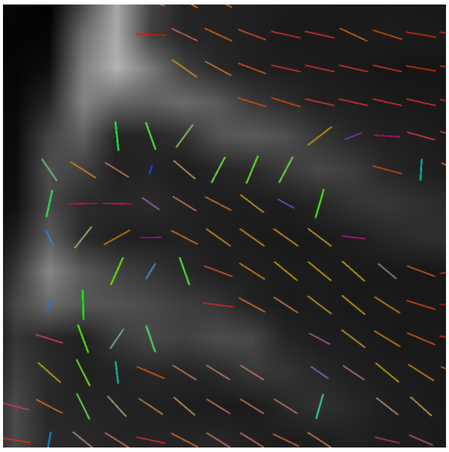

Figure S2. CSD, predominant fixels.

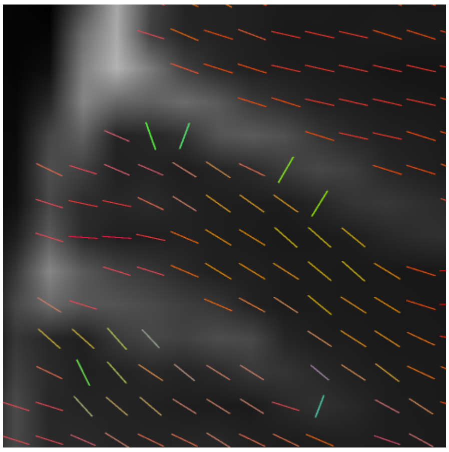

Figure S3. LNNN, predominant fixels.

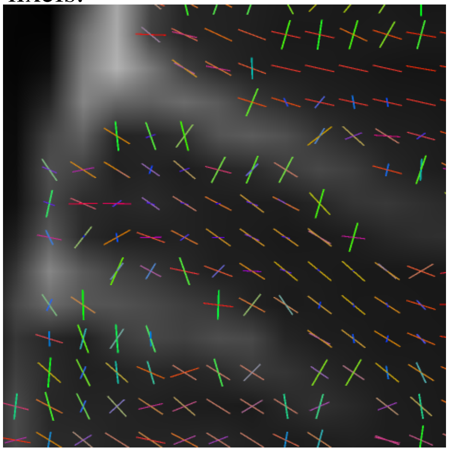

Figure S4. CSD.

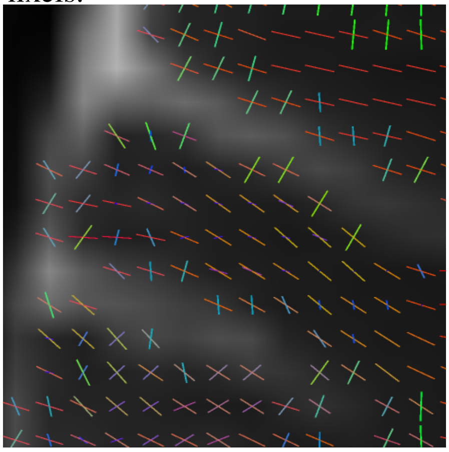

Figure S5. LNNN.

Figure S6. Results on Stanford HARDI image. Zoom in at a gyral blade. Upper row: predominant fixels. Lower row: first and second most predominant fixels.
