# Supplementary material for: Light-weight neural network for intra-voxel structure analysis
Source: Front Neuroinform. 2024 Sep 9;18:1277050. doi: 10.3389/fninf.2024.1277050 (PMC11417038; doi:10.3389/fninf.2024.1277050)
Supplement: Supplementary file 2 [file Data_Sheet_2.PDF]

1.2 Tables

	n_2				
n_1	**256**	**512**	**768**	**1024**	**1536**
**128**	7.17e-06	7.14e-06	7.21e-06	7.25e-06	6.84e-06
**256**	7.03e-06	7.01e-06	7.04e-06	7.11e-06	6.70e-06
**512**	6.98e-06	*6.96e-06*	6.98e-06	7.14e-06	6.58e-06
**768**	6.99e-06	6.92e-06	7.01e-06	6.98e-06	6.54e-06
**1024**	7.02e-06	6.92e-06	7.02e-06	7.19e-06	6.61e-06

Table S1: MSE performance of neural networks with a different number of neurons on each of the two layers. Models trained with $W10$ labels.

	n_2				
n_1	**256**	**512**	**768**	**1024**	**1536**
**128**	0.42	0.77	1.13	1.48	2.19
**256**	0.74	1.36	1.98	2.60	3.83
**512**	1.40	*2.54*	3.68	4.82	7.11
**768**	2.06	3.72	5.39	7.05	10.38
**1024**	2.71	4.90	7.09	9.28	13.66

Table S2: Millions of trainable parameters in networks with different sizes in the first and second layers.
