# Supplementary material for: Light-weight neural network for intra-voxel structure analysis
Source: Front Neuroinform. 2024 Sep 9;18:1277050. doi: 10.3389/fninf.2024.1277050 (PMC11417038; doi:10.3389/fninf.2024.1277050)
Supplement: Supplementary file 3 [file Data_Sheet_3.PDF]

Supplementary Material

1 SUPPLEMENTARY TABLES AND FIGURES

1.1 Figures

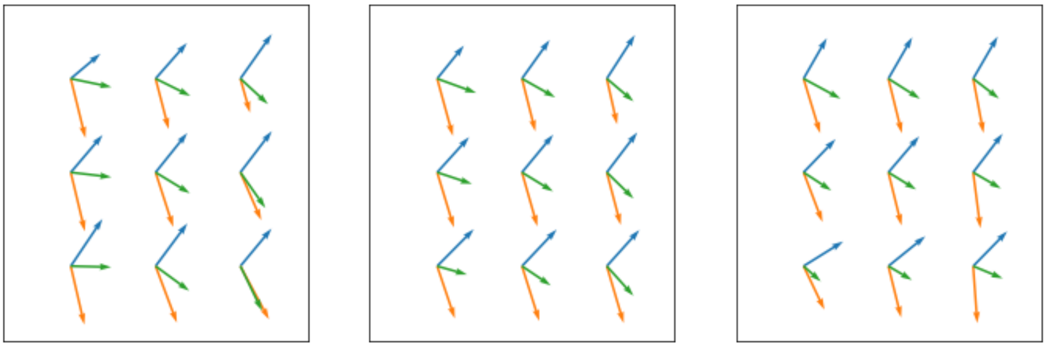

Figure S1. Fiber orientations in a neighborhood. Left to right correspond to the top to bottom of the 3D cube. The datum shown here was extracted from the training dataset.
