# Supplementary material for: Light-weight neural network for intra-voxel structure analysis
Source: Front Neuroinform. 2024 Sep 9;18:1277050. doi: 10.3389/fninf.2024.1277050 (PMC11417038; doi:10.3389/fninf.2024.1277050)
Supplement: Supplementary file 4 [file Data_Sheet_4.PDF]

Supplementary Material

1 SUPPLEMENTARY DISCUSSION ABOUT EMD

Distributions and peaks.

In this first example, we compare a Dirac delta over any other distribution. This is the case when comparing an fODF and a single orientation.

Let $\mathbf{a}$ be a vector with entries

$$\mathbf{a_i} = \begin{cases} 1, & \text{if } i = k \\ 0, & \text{otherwise} \end{cases},$$

and $\mathbf{b}$ a probability vector. Then, the first restriction $P\mathbb{I} = \mathbf{a}$ imply that $P_{ij} = 0$ for all $i \neq k$, and the second restriction, $P^\top \mathbb{I} = \mathbf{b}$, imply that $P_{kj} = \mathbf{b}_j$. Consequently,

$$EMD(\mathbf{a}, \mathbf{b}) = \sum_j C_{k,j} \mathbf{b}_j. \tag{S1}$$

That is, the sum of the distances between the elements of $\mathbf{b}$ and $\mathbf{a}_k$, weighted by $\mathbf{b}$.

Translations.

One interesting decomposition of the EMD is that given a probability vector $\mathbf{a}$, and considering the EMD between the support of $\mathbf{a}$ and the same elements translated by τ, the EMD for Euclidean cost can be decomposed as:

$$EMD(\mathbf{a}, T_\tau(\mathbf{a})) = ||\sqrt{\mathbf{a}}\tau||^2. \tag{S2}$$

2 SUPPLEMENTARY TABLES AND FIGURES

2.1 Figures. Inner product between the inferred fixels with CSD and LNNN

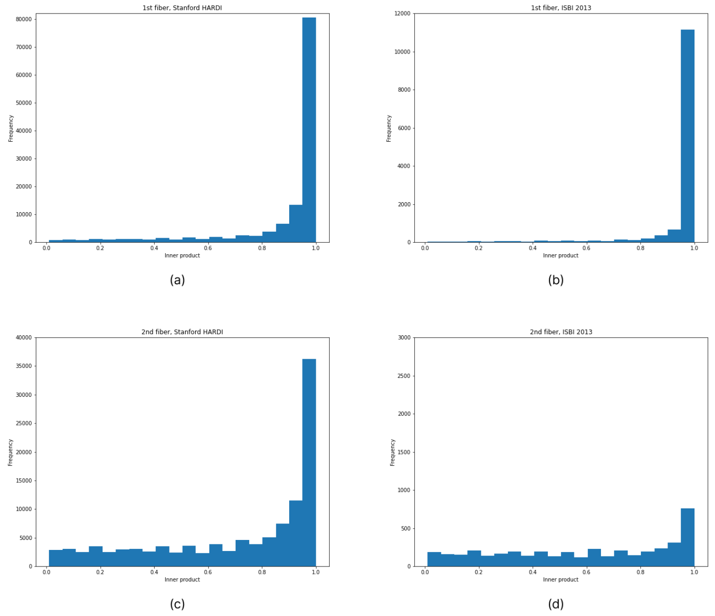

Figure S1. We observe histograms of the inner product of the estimations produced by CSD and LNNN in their first two dominant fibers. In figure **(a)** and **(b)** we observe the behaviour of both methods in the first fiber with the real data (Stanford HARDI, **(a)**) and the synthetic data (ISBI 2013, figure **(b)**). We observe the same comparative in **(c)** and **(d)** for the ISBI 2013 synthetic data. Both methods differ very similar in both datasets, which might indicate a similar performance of LNNN and CSD in both datasets.

Our hypothesis is that metrics based on real data could reflect that the LNNN method is more accurate than the CSD. This risky hypothesis is supported by our results on synthetic data and the analysis of this Figure. It shows that the distributions of the inner product between the estimate first and second fixels with CSD and LNNN follow a similar behavior independently of their origin: real or synthetic data. Then, we might expect the performance of fixed estimation methods on real data to follow a similar behavior.
